# A Review of Hypothesized Determinants Associated with Bighorn Sheep (*Ovis canadensis*) Die-Offs

**DOI:** 10.1155/2012/796527

**Published:** 2012-03-29

**Authors:** David S. Miller, Eric Hoberg, Glen Weiser, Keith Aune, Mark Atkinson, Cleon Kimberling

**Affiliations:** ^1^P.O. Box 2786, Loveland, CO 80539-2786, USA; ^2^U.S. National Parasite Collection, ARS, USDA Animal Parasitic Diseases Laboratory BARC, East 1180 10300 Baltimore Avenue, Beltsville, MD 20705, USA; ^3^Caine Veterinary Teaching Center, College of Agriculture and Life Sciences, University of Idaho, 1020 East Homedale Road, Caldwell, ID 83607, USA; ^4^Montana Fish Wildlife and Parks, 1400 South 19th Avenue, Bozeman, MT 59715, USA; ^5^Wildlife Conservation Society, 2023 Stadium Drive, Suite. 1A, Bozeman, MT 59715, USA; ^6^Wildlife Conservation Society, 2300 Southern Boulevard, Bronx, NY 10460, USA; ^7^Department of Clinical Sciences, College of Veterinary Medicine and Biomedical Sciences, Colorado State University, Fort Collins, CO 80523, USA

## Abstract

Multiple determinants have been hypothesized to cause or favor disease outbreaks among free-ranging bighorn sheep (*Ovis canadensis*) populations. This paper considered direct and indirect causes of mortality, as well as potential interactions among proposed environmental, host, and agent determinants of disease. A clear, invariant relationship between a single agent and field outbreaks has not yet been documented, in part due to methodological limitations and practical challenges associated with developing rigorous study designs. Therefore, although there is a need to develop predictive models for outbreaks and validated mitigation strategies, uncertainty remains as to whether outbreaks are due to endemic or recently introduced agents. Consequently, absence of established and universal explanations for outbreaks contributes to conflict among wildlife and livestock stakeholders over land use and management practices. This example illustrates the challenge of developing comprehensive models for understanding and managing wildlife diseases in complex biological and sociological environments.

## 1. Introduction

Effective management and conservation of wildlife populations can be undermined by multiple causes. These include decreased and altered habitat and other direct anthropogenic effects, climate change, competition and predation from nonnative wildlife and domestic species, demographic challenges associated with small populations, multiple, incompatible management objectives for sympatric species or their habitat, and exposure to native and exotic infectious agents [1–4]. The consequences and interactions of these variables are difficult to understand and predict, and may vary by circumstances. This uncertainty, particularly when it occurs in complex sociological environments where stakeholders have differing values and objectives, presents substantial challenges for decision makers. In such uncertain environments, the absence of data and differing values can result in polarized debate among stakeholders. It can also serve as an impediment to the acquisition of data that would contribute to effective management. Respiratory disease outbreaks in bighorn sheep (*Ovis canadensis*) illustrate the challenge of identifying and managing disease in valued wildlife populations, where stakeholder perceptions and values clash [5].

Bighorn sheep are highly valued for recreational, ecological, philosophical, spiritual, and other reasons [6]. Bighorns have experienced a population decline of two orders of magnitude subsequent to 19th century settlement of western North America [7]. This decline has been attributed to a combination of human activities, such as overhunting, domestic livestock grazing, introduced infectious agents, and displacement from range and migratory paths. While translocations and other management activities have resulted in partial recovery of populations and numbers, die-offs have seriously undermined bighorn sheep recovery efforts [8]. Moreover, uncertainty regarding the agents, reservoirs, and causes of outbreaks has contributed to stakeholder polarization [5, 9]. Recent die-offs at multiple locations during the winter of 2009-2010 highlight the need to identify causes and potential management strategies for respiratory disease outbreaks in bighorn sheep [10].

Research to date has largely focused on identifying an agent and reservoir responsible for causing bighorn die-offs. Early research on bighorn respiratory disease die-offs focused on lungworm-pneumonia complex, due to protostrongylid lungworms (*Protostrongylus* spp.) that are likely indigenous parasites of bighorn sheep and other free-ranging caprines [11, 12]. More recent research has focused largely on Pasteurellaceae with the hypothesis that domestic sheep (*O. aries*) serve as reservoirs for an infectious agent or agents that is (are) fatal in bighorn sheep, as well as in response to policy-driven arguments against this hypothesis [13–16]. However, placing research on agents and reservoirs in context is difficult, particularly for data collected under captive conditions. One contextual challenge is the inconsistency in agents associated with different outbreaks [17–19]. In addition, contrasting models of transmission have been proposed. Some outbreaks subjectively appear to be propagated epidemics [19, 20], possibly due to a single, novel infectious agent that was recently introduced into a population. In contrast, web of causation [21] models represent a more holistic perspective that considers multiple environmental, host, and agent determinants of disease and their interaction. Web of causation models are similar to those accepted for domestic livestock models of “shipping fever” [22–26], and leave open the potential for endemic agents to cause outbreaks with certain combinations of determinants. The potential for endemic agents to favor outbreaks, or to sporadically cause increased morbidity or mortality that is perceived as an outbreak, has been less considered than the introduction of novel agents to naïve populations. Resolution of these issues, in part, will contribute to development of management strategies and resolution of contention regarding land management policies for domestic and bighorn sheep. Full resolution will likely require sociological approaches to resolve differing values for land use among stakeholders.

The objective of this paper is to critically consider the relative role of various factors in limiting bighorn sheep populations, with an emphasis on outbreaks and infectious agents. Since there is uncertainty as to whether endemic agents can sporadically cause outbreaks, this review will first consider environmental and host determinants of disease. This broad-based approach also helps to place the impact of infectious disease in context with other population-limiting factors. It will not be assumed that these determinants are temporally and spatially constant. This approach is consistent with the use of multiple working hypotheses for scientific investigations [40]. Practical and sociological considerations that inhibit the resolution of biological questions will be acknowledged where relevant. While the available data does not support quantitative assessment of risk factors for die-offs, this paper can serve as a starting point for development of new approaches for addressing the challenge of bighorn sheep respiratory disease die-offs and management of infectious diseases in wildlife populations.

## 2. Determinants Limiting Free-Ranging Bighorn Sheep Populations

### 2.1. Direct Mortality

 Historic and current causes of direct mortality serve as a point of comparison for mortality due to sporadic outbreaks, and their relative impact may change as circumstances vary. Causes of direct mortality to bighorn sheep include hunting and predation (Table 1). Hunting is considered a substantial cause of bighorn sheep population declines in the 19th and early 20th centuries [7]. Current regulations largely minimize mortality due to hunting, but must be responsive to changing conditions, due to the potential for localized or range-wide adverse impacts. Stakeholders are largely supportive of sustainable yields for bighorn sheep.

 Predation has been a concern in several regions throughout bighorn sheep range (Table 1). Predation by puma (*Felis concolor*) may limit bighorn sheep in locations where predator populations are largely supported by sympatric native or domestic ruminant populations [33–35, 38]. It has been proposed that the impact may be greatest where pumas specialize on small populations of bighorn sheep [29, 30]. However, predation losses may be compensatory and may not generally limit bighorn sheep populations [41]. Indirect impacts of predation, such as predator avoidance behaviors, might predispose bighorn sheep to disease and die-offs, but this has not been established. Predator control programs could limit bighorn mortality in small, demographically vulnerable populations, where this strategy is acceptable to stakeholders.

### 2.2. Environmental Determinants

 Environmental determinants are known to directly or indirectly affect the susceptibility of animals to disease [22] and have been proposed to be limiting factors for bighorn sheep populations (Table 2). Combinations of extreme cold, heavy snow, or other adverse conditions may sporadically compromise bighorn sheep health and predispose to infectious disease die-offs, but are rare direct causes of mortality [42, 43]. Escape terrain is a temporally stable feature that probably characterizes where bighorn sheep can avoid predation and persist [44], but is unlikely to vary in space and time [45, 46]. In contrast, historic bighorn sheep range and migratory pathways to seasonal ranges are not temporally stable, due to anthropogenic activities [47, 48]. Regardless of whether physical environmental characteristics vary temporally or spatially or whether there are infectious disease concerns, understanding the impact of these characteristics on bighorn sheep carrying capacity is critical for designing management strategies for long-term persistence. There is a need to determine whether bighorn sheep populations at or below carrying capacity are less prone to outbreaks of disease.

Bighorn sheep carrying capacity is a dynamic function of food and water resources that vary seasonally and with weather patterns [49–52]. Recognition of this and its impact on animals is analogous to the basics of animal husbandry, where meeting animal food and water requirements is important for maximizing animal health and fecundity and minimizing disease. It is also a core wildlife management principle [1]. Proximity to free-standing water appears to be a good predictive variable for bighorn sheep in arid regions, much as provision of water represents basic animal husbandry [53]. However, the sequential hypothesis that increasing water availability will increase desert bighorn sheep carrying capacity, improve animal health, or reduce the risk of transmissible diseases has not been consistently supported [54–58]. Studies with small numbers of experimental units limit resolution of this issue, as do confounding variables that are inconsistently reported, including the quantity and timing of precipitation, amount of forage available, and forage water content. Development of artificial water sources represents a logical and potentially popular resolution to perceived conservation needs for bighorn, but confirmation of the relative risks and benefits is needed.

 Precipitation's effect on the quantity and quality of forage available to bighorn sheep is a range-wide factor that can impact the nutritional health of bighorn sheep and sympatric species (Table 2). It has been reported that the quantity of forage available to bighorn sheep, recruitment, and carrying capacity are positively correlated with the quantity of precipitation [59–64]. Precipitation and forage production have been speculated to be proportionate to bighorn sheep resistance to disease [49, 65]. Furthermore, forage nutritional quality (i.e., digestible energy, protein, and minerals) varies spatially and with the timing of precipitation, and this may not coincide with bighorn sheep nutritional requirements at specific stages in their annual cycle. This is because plant community structure and the nutritional quality of different stages of plant growth vary based on when precipitation occurs during the growing season [60, 63, 66–68]. While little is known of bighorn sheep nutritional requirements, based on elk models [69], small differences in forage digestible energy that are not visibly detectable may affect bighorn sheep fecundity and survival. These differences in digestible energy are likely intertwined with other nutritional requirements, such as those hypothesized for selenium, protein, and minerals [63, 70, 71]. An additional confounder is where forest succession or other advanced plant seral stages limit the presence of herbaceous plants [72, 73]. Greater clarity on the impact of precipitation on bighorn sheep forage quality and quantity may identify indices that can be used to identify periods of nutritional compromise, such as during periods of drought. These periods may represent times of increased risk for development of disease from endemic agents, or animal movements that favor increased exposure to novel agents. While precipitation cannot be managed, historical and predicted patterns can be incorporated into management plans, and strategies such as controlled burns [74–76] might be used to modify forage nutritional quality. However, the consequences of temporal variability in forage can be difficult to communicate. This may create obstacles to stakeholder acceptance of sporadic die-offs or other stochastic events that undermine achievement of management objectives.

Bighorn sheep nutrition can also be affected by competition for forage because of density-dependant effects from conspecifics and sympatric species (Table 2). Competition with domestic sheep is recognized for its potential to compromise long-term bighorn sheep persistence, especially in the context of climate change [77]. However, overgrazing by other domestic species, native ungulates, and high bighorn sheep populations have also been described in case reports for almost a century and in a recent computer simulation [3, 72, 78]. These effects may be seasonal, based on reports of adverse competitive effects that are specific to bighorn sheep winter range, yet have long-term impacts on population fecundity and mortality rates. Socially mediated competitive effects may also exist, based on reports of bighorn avoidance of domestic species under field conditions, and decreased maternal care and neonatal survival at high bighorn sheep population densities [59, 79–81]. Consequently, there is a need to quantify the carrying capacity and social impacts of multiple sympatric species on rangeland. Even where this information can be established, effectively addressing management interests that may conflict remains a challenge. For example, an agricultural enterprise could pose a threat to bighorn sheep health while concurrently preventing development of critical habitat used by bighorn sheep and other wildlife species [82]. Such dilemmas can be difficult to overcome for both biological and sociological reasons, particularly where viewpoints are polarized.

Environmental determinants of disease are well accepted as being relevant to animal health [22]. Consequently, it would not be surprising if there are environmental determinants of disease that favor bighorn sheep outbreaks. While some environmental determinants may be distributed throughout bighorn sheep range, spatial and temporal variation occurs. Understanding this variation may be useful for developing predictors of outbreaks or increased levels of chronic disease. Greater attention to the impacts of precipitation and inter-/intra-species competition on forage quantity or quality may reveal predictors that favor introduction of novel agents into bighorn sheep populations or outbreaks from endemic agents. Consequently, there is a need to develop methods for evaluating bighorn sheep carrying capacity as it varies with time. An understanding of environmental determinants of disease will be required for development of management strategies that can respond to anthropogenic and climatic impacts on bighorn sheep habitat.

### 2.3. Host Determinants

Host determinants are known to directly or indirectly affect the susceptibility of animals to disease [22]. Knowledge of host determinants of disease for bighorn sheep is incomplete. These determinants can be considered as extrinsic, such as previously discussed for nutrition, or intrinsic. Low variation in the major histocompatibility complex has been hypothesized as an intrinsic determinant that could result in a high susceptibility to infectious disease in bighorn sheep, but data does not support this hypothesis [123, 124]. Immune suppression due to elevated cortisol responses to external stressors has also been hypothesized to be an intrinsic determinant of disease [93]. However, standardized necropsy protocols for investigating the “stress” hypothesis have not been developed and applied to outbreaks. In addition, although noninvasive, antemortem evaluation of cortisol levels can be conducted with fecal assays, few studies use this method to quantify the cortisol responses of free-ranging bighorn sheep to potential stressors [125, 126]. It is conceivable that elevated cortisol levels from rut activity could suppress bighorn sheep immune systems and be a determinant of disease for some outbreaks [43]. This is analogous to mortality and reduced resistance to disease in male *Antechinus stuartii, *a small marsupial, due to elevations in corticosteroids during the breeding season [127]. However, data on cortisol responses to rut, inter- and intra-specific interactions, anthropogenic activities, and other potential stressors is needed to fully evaluate the “stress” hypothesis. Such work will need to distinguish between cortisol responses, social behavior that facilitates transmission of infectious agents, and other host factors that may favor development of disease. Although little work has been done to establish host determinants as proximate or ultimate causes of outbreaks, identification of these determinants may provide a means of testing some hypotheses or as indices for identifying populations at risk of outbreaks.

### 2.4. Agent Determinants

 Agents may act as primary pathogens, or cause opportunistic infections under some combinations of agent, environmental, or host determinants. A practical challenge that exists for identifying agent determinants of disease is their presence in both healthy and diseased bighorns [17, 18, 128]. A further challenge is the absence of consistently used definitions of subclinical infection and disease. Because baseline data is generally absent and logistic constraints can limit collection of useful diagnostic samples during an outbreak, it can be uncertain as to whether a given agent acts as a primary pathogen, an opportunistic pathogen, or is an incidental isolate. Primary pathogens are most likely to be novel agents that are introduced into a naïve population. If an agent causes opportunistic infections, it implies that the host is compromised due to other agent, host, and/or environmental determinants, and the agent may be endemic in the population. A corollary is that compromised hosts are also more vulnerable to developing disease from many agents. Agents that typically cause chronic or low-prevalence infections can be the cause of outbreaks if other determinants favor an increase in the prevalence and/or severity of disease. These distinctions are important because different management approaches may be needed to address the introduction of novel agents and opportunistic infections. It is also important to recognize that a given agent may not always be responsible for outbreaks or consistently act as either a primary or opportunistic infection, due to spatial or temporal variation, or the influence of other determinants.

#### 2.4.1. Parasites

 Bighorn sheep harbor a number of ecto- and endo-parasites [129]. Based on general animal models, these agents may act as primary pathogens or increase susceptibility to disease from other agents. Sheep scab (*Psoroptes* spp.) is an ectoparasite that has been reported range-wide and was first associated with bighorn sheep die-offs during 19th century settlement of bighorn sheep range [130–132] (Table 3). While early reports are consistent with the hypothesis that these outbreaks resulted from the introduction of novel parasites to bighorn sheep, probably from domestic sheep, the ecology, reservoirs, and taxonomy of bighorn sheep *Psoroptes* spp. have not been fully resolved [133–135]. *Psoroptes* spp. is of interest to the livestock industry because it is a reportable agent, but infestations of domestic sheep may be subclinical, and clinical signs may be a function of an individual's health status, reproductive stage, and immunity to the mites [136–138]. This may be similar in free-ranging bighorn sheep, where subclinical infestations, self-resolution of infestations without human intervention, and outbreaks associated with rut or drought conditions have been reported [139–142]. An outbreak, where high animal densities and drought were described, illustrates the challenge in distinguishing between compromised animal health due to competition for nutrition, density-dependent “stress” responses of the host, density-dependent transmission of an agent, and other factors [143]. However, in sum, these observations suggest that multiple environmental, host, and agent determinants may determine the clinical course of bighorn sheep *Psoroptes *spp. infestations. While it may be speculated that these determinants may also render bighorn sheep vulnerable to respiratory disease, a link between these two has not been established. Nevertheless, the potential for range-wide impacts and disease to be favored by changing conditions illustrates the importance of understanding how combinations of environmental, host, and agent factors may favor the transition from subclinical and/or low-prevalence disease to outbreaks.

*Protostrongylus* spp. (lungworm) are native endoparasites of bighorn sheep that are found range-wide, except under xeric conditions where gastropod intermediate hosts required for transmission are absent (Table 3) [144, 145]. *Protostrongylus* spp. have been associated with all-age die-offs (Table 3), and are also a source of summer verminous pneumonia in 3–6-w-old lambs [129, 146, 147]. Verminous pneumonia of lambs is the consequence of numerous L_3_ larvae synchronously maturing in the lungs. However, all-age die-offs may be due to opportunistic bacterial infections that are secondary to lungworm lesions, based on isolation of multiple bacterial species from pneumonic lungs, histopathology, and recovery of lungworm from bighorn sheep without clinical disease [18, 27, 113, 148–150]. The absence of apparent disease in free-ranging bighorn sheep experimentally inoculated with *P. stilesi* and *P. rushi*, as well as observations of bighorn sheep with respiratory disease and low pulmonary burdens of lungworm, raises further doubt about the role of lungworm as primary pathogens [129, 151–154]. In addition, while the number of pulmonary lungworm present is correlated with precipitation, presumably due to favorable conditions for gastropod-intermediate hosts, lamb recruitment is also highest under such conditions [50, 59, 98, 148, 155]. This suggests that animals with good nutrition can moderate the effects of lungworm infections. Furthermore, while administration of anthelmintics is a logical response to lungworm infections, a study of multiple populations that used a crossover design indicated that this strategy is not efficacious for improving lamb recruitment in free-ranging bighorn sheep populations [156]. Thus, a simple, invariant relationship between lungworm and disease does not appear to exist for bighorn sheep. Consequently, other determinants, such as competition from high densities of bighorn sheep, native ungulates, and domestic livestock, may be factors predisposing to outbreaks [7, 113, 118].

*Muellerius capillaris*, a lungworm of domestic sheep that has become established in some bighorn sheep populations, may cause disease under some conditions [125, 171, 191]. Similar historic introductions in other settings have been recognized to be responsible for a mosaic landscape of native and introduced parasite species [192]. *Protostrongylus* spp. and *Muellerius capillaris* illustrate the challenges of discriminating between proximate and ultimate causes of disease, the importance of rigorous study design, the potential for nonnative agents to become established and cause disease in bighorn sheep populations, and the potential for agents responsible for clinically mild and/or low prevalence infections to contribute to outbreaks when favored by other determinants. Further, recent studies have indicated that the presence of dorsal-spined larvae in feces is not always indicative of infection by *M. capillaris*, but may involve L_1_ (first stage larvae) of muscleworms, specifically *Parelaphostrongylus odocoilei* [191, 193]. Significantly, the potential of *P. odocoilei* as disease agents in free-ranging wild-sheep (thin horn, *Ovis dalli*) has been demonstrated [194], although the extent of distribution for these parasites among populations of bighorn is undetermined.

Lungworm and muscleworms also illustrate methodological limitations that exist for agent surveillance in bighorn sheep [195–198]. Baermann analysis of feces has commonly been used as an antemortem, semiquantitative method for determining the number of L_1_ per gram of feces (LPG) in bighorn sheep; standard analyses have now been superseded by the modification termed “beaker Baermann” which has superior recovery [199]. This approach is similar to fecal analyses of domestic livestock for endoparasite management in pasture environments [137]. However, whereas the domestic livestock parasites of interest have direct life cycles, lungworm and muscleworms have a gastropod intermediate host. Consequently, LPG is a poor index of transmission, as it requires temporally consistent fecal shedding of L_1_, proportionate fecal L_1_ infection and development to L_3_ in gastropods, and proportionate consumption of L_3_-infected gastropods by bighorn sheep to be predictive for parasite transmission. In addition, LPG has not been correlated with pulmonary lesions and is therefore not a valid index of body burdens of parasites [150, 155]. Another confounding factor is that until recently, the L_1_ of protostrongylids found in feces could not be definitively identified. Currently it is possible to separate all North American genera and species based on diagnostic molecular sequences (e.g., [200]). Use of LPG as an index of parasite “load” or as an index of transmission, however, does not have a strong biological basis, yet has been used in bighorn sheep parasitological studies due to an absence of alternative tools.

 Scabies and lungworm illustrate agents with potential range-wide impacts on bighorn sheep. However, their role as proximate or ultimate causes of outbreaks may vary; they can be present without clinically apparent disease, yet may have a role in some outbreaks. This picture is further complicated by the concurrent presence of other parasites that may reduce animal's resistance to disease (e.g., coccidia), or that cause localized morbidity (e.g., nose bot flies, *Oestrus ovis*) [89, 201–203]. In addition, parasites can serve as vectors for other infectious agents of bighorn sheep [204]. Consequently, parasite's potential to contribute to outbreaks merits consideration, whether they are native agents, exotic agents, are intermittently reintroduced into populations, or are endemic. Parasites may be a particular concern for migratory populations that have become sedentary, due to the potential for high animal densities and/or extended exposure that favors transmission. In addition, other host-parasite systems illustrate the potential for climate change to result in expansion of parasite's range or altered transmission dynamics [205–207]. Consequently, use of improved methodology and study design to identify host, environmental, and agent interactions is needed for the development of short- and long-term bighorn sheep management strategies.

#### 2.4.2. Bacteria

 Bacteria are commonly a component of respiratory disease in many domestic and nondomestic species. They can be either primary or opportunistic infections. Early reports hypothesized that bacteria were opportunistic infections of bighorn sheep that occurred when favored by host, environmental, or agent determinants [113, 157, 166]. This is similar to the shipping fever model of respiratory disease in domestic livestock. The shipping fever model views pasteurellosis as an opportunistic disease that results when endogenous Pasteurellaceae colonize the lungs of livestock compromised by different combinations of infectious agents, host and environmental determinants [23, 24, 208–210]. This model arose subsequent to the failure of single-agent experiments to provide an explanation for shipping fever. Recognition of the multifactorial nature of shipping fever resulted in the development of multiple, potentially concurrently used, management strategies to reduce its prevalence and severity in livestock.

The Pasteurellaceae are a heterogenous group that has experienced many taxonomic changes. A biovariant system of classifying Pasteurellaceae for wildlife work has been developed due to isolates that were not typable using conventional serologic classification systems [172], but utilization of genotype-based methods may be more appropriate for some research questions [211]. *Mannheimia haemolytica, Pasteurella multocida,* and* Bibersteinia* (formerly *Pasteurella*)* trehalosi* have been isolated from pneumonic and healthy bighorn sheep over much of their range, although experimental work over the past half century has largely focused on *M. haemolytica* from presumptive domestic sheep reservoirs [14, 128, 173, 212, 213] (Table 3). Evidence consistent with domestic sheep Pasteurellaceae acting as a primary pathogens of bighorn includes isolation of Pasteurellaceae from pneumonic bighorn sheep during outbreaks, outbreaks in free-ranging and captive bighorn sheep following “contact” with domestic sheep and goats, pneumonia in bighorn sheep caused by experimental inoculations of isolates native to domestic sheep, *in vitro* evidence of a cellular basis for bighorn sheep's particular sensitivity to disease from *M. haemolytica*, and experimental evidence for transmission from domestic sheep causing disease in bighorns [15, 19, 151, 214–216]. However, it has not yet been demonstrated that any Pasteurellaceae are found more often in animals with disease than in healthy animals. In addition, Pasteurellaceae of apparently healthy domestic and bighorn sheep under field conditions can be similar, and there is evidence for interspecies “contact” occurring under field conditions without disease resulting [128, 174, 217, 218]. The similarity of Pasteurellaceae among bighorn and domestic sheep may represent the historic introduction and establishment of novel strains in populations, and it is conceivable that this could predispose animals to disease. Thus, there is a need to identify Pasteurellaceae that are associated more with pneumonic bighorn sheep than with apparently healthy animals. Alternatively, it is possible that pasteurellosis is an opportunistic infection that results when favored by other host, environmental, or agent determinants. A host determinant hypothesized to favor pasteurellosis is elevated levels of corticosteroids in response to external stressors [93, 219]. There is a need to clarify the degree to which Pasteurellaceae act as primary pathogens, and whether other determinants are needed to favor respiratory disease outbreaks.

Domestic livestock, and possibly bighorn sheep, can experience a range of clinical severity to Pasteurellaceae. This includes self-resolution of infections. Histopathological evidence for resolution of bronchopneumonia suggests that bighorn sheep may experience a similar range of clinical severity and self-resolution [18, 181]. There is also evidence that pasteurellosis can cause chronic, sporadic disease that can negatively impact bighorn sheep populations, as opposed to acute outbreak impacts [41]. Isolation of bacteria such as *Arcanobacterium* (formerly* Corynebacterium* and *Actinomyces*)* pyogenes* from pneumonic bighorn sheep, and isolation of *Histophilus somni*, and other Pasteurellaceae from apparently healthy animals, are consistent with pasteurellosis as an opportunistic infection that originates from endogenous, commensal bacteria [17, 113, 153, 157, 220]. If this is true, other commensal bacteria may cause disease in the absence of Pasteurellaceae.

 Strategies considered for mitigating the effects of pasteurellosis in bighorn sheep include administration of antibiotics, vaccines, and quarantine establishment. Although biomedical approaches are established and well-perceived strategies, there is a lack of demonstrated vaccine or medication efficacy to date [18, 112, 221–223]. Several explanations are possible for this, but the dearth of published data supporting the efficacy of vaccines for pasteurellosis in domestic ruminants under field conditions illustrates the challenges of developing products and documenting efficacy [26, 224, 225]. Alternatively, 14.5 km buffers between bighorn and domestic sheep have been employed as a quarantine strategy to minimize interspecies transmission of Pasteurellaceae [226], although long-distance movements by both species can undermine this approach. Nevertheless, in principle, buffers are useful for multiple reasons, such as minimizing interspecies transmission of multiple agents, minimizing competition for forage, mitigating against potential stress responses of bighorn sheep to domestic animals, and potentially for other reasons, particularly for small-, high-risk bighorn populations. Management of environmental and host determinants may be effective alternatives to biomedical strategies [21] if validated, but delayed effects and indirect mechanisms of action may be difficult to explain for obtaining stakeholder and public support.

*Mycoplasma ovipneumoniae* has been considered both a primary pathogen and as a predisposing agent for secondary pasteurellosis in domestic ruminants, particularly in lambs <1 y [227, 228]. However, much as with Pasteurellaceae, *Mycoplasma* spp. appears to be a common commensal of domestic sheep, and it is believed that disease may primarily occur when favored by certain combinations of host, environmental, and agent determinants [229]. It is not clear to what extent *Mycoplasma* spp. are responsible for outbreaks in bighorn sheep (Table 3). *Mycoplasma ovipneumoniae* has been associated with bronchopneumonia in free-ranging bighorn sheep, and could be an explanation for depressed lamb recruitment in years following outbreaks [179]. However, while *Mycoplasma* spp. may be an agent that could be introduced into naïve bighorn sheep populations from domestic sheep, the percentage of free-ranging pneumonic bighorn sheep with evidence of *Mycoplasma* spp. varies from 7–55%, a bighorn sheep that closely associated for several months with domestic livestock with *Mycoplasma* spp. was apparently uninfected at necropsy, and limited experimental inoculations of bighorn sheep lambs failed to result in fatal respiratory disease [18, 19, 128, 179]. Thus, while *Mycoplasma* spp. may contribute to bighorn sheep outbreaks, multiple determinants may be required for this to occur, much as appears to be the case for Pasteurellaceae. A caveat is that *Mycoplasma* spp. is not routinely tested for, and many laboratories have a limited diagnostic capability for this agent. Therefore, our understanding of whether *Mycoplasma* spp. is partly or generally responsible for bighorn sheep outbreaks may be limited by methodology. In addition, if *Mycoplasma* is demonstrated as an important determinant of bighorn sheep respiratory disease, the limited options for efficacious treatment or vaccination of domestic livestock suggest that biomedical management options would not be available for bighorns in the near future.

Multiple bacterial species have been associated with respiratory disease in bighorn sheep, much as has been documented for other species. Outbreaks of bacterial ocular disease in bighorn sheep illustrate the potential for bacterial pathogens to be transmissible, and for livestock to be a reservoir for transmissible bacterial pathogens [178, 180]. However, the degree to which endogenous versus transmissible bacteria generally contribute to disease is unclear, reservoirs are uncertain, factors affecting the dynamics of transmissible agents are undetermined, and the degree to which specific bacteria contribute to specific outbreaks in free-ranging populations or in general needs clarification. This uncertainty is due to constraints in methodology and study designs (predominantly case reports and cross-sectional studies) that limit the inference possible from historic data. Biomedical approaches for managing disease in domestic animals have been successful, but outside of rabies, examples of where these approaches have been effective in wildlife are limited. Furthermore, rigorously demonstrating product efficacy in bighorn sheep will require a long timeline. Based on shipping fever models of respiratory disease in livestock, development of multiple strategies for management of host and environmental determinants of bighorn sheep disease is needed for current conditions and in response to anthropogenic and climate changes.

#### 2.4.3. Virus

 Viral respiratory pathogens have been associated with bighorn sheep outbreaks, and there is serologic evidence that animals can be infected and recover from these agents (Table 3)[128, 182, 185]. This is similar to the role of viruses as primary pathogens or as agents that predispose livestock and other species to opportunistic bacterial infections [24, 208, 230]. This poses many of the same questions that exist for parasitic and bacterial infections of bighorn sheep. Method and study design limitations present challenges for determining: reservoirs for viral agents; whether viral agents act as primary respiratory pathogens; or whether other agent, host, or environmental codeterminants are required for disease to develop.

A high seroprevalence of antibodies to agents such as parainfluenza-3 and bovine respiratory syncytial virus in some bighorn sheep populations suggests that infections may be common and clinically mild or incidental [128, 184, 186]. However, the potential for disease to develop when favored by other determinants must be considered, particularly if these are nonnative agents that have become established in bighorn sheep populations. In addition, an increase in disease associated with nonrespiratory viral agents is a consideration if transmission dynamics are altered by increased population densities, climate change, or other determinants [187, 188]. Longitudinal studies of multiple populations will likely be required to determine the degree to which viruses and other determinants contribute to respiratory disease outbreaks generally or in specific instances, as well as whether disease is a density-dependent phenomena. Where viruses are primary pathogens or are a part of multiple agent infections, viral vaccines of demonstrated efficacy may decrease the prevalence and/or severity of infections, if livestock models can be applied to bighorn sheep [231].

### 2.5. Mixed Infections

 Multiple agents have been associated with bighorn sheep outbreaks. Some reports have indicated that multiple agents were concurrently responsible for outbreaks as mixed infections, with environmental or host determinants implicated as ultimate causes [18, 90, 113, 120]. If this is true, environmental and host determinants may need to be targeted to effectively manage outbreaks, and the specific strategies that are most effective may vary spatially and temporally. Knowledge of agents, modes of transmission, and mechanisms of disease may not be required to develop effective host and environmental management strategies for reducing respiratory disease in bighorn sheep. This perspective is illustrated by risk factor analyses that demonstrated strategies for decreasing cholera and lung cancer in humans, even though the agents and mechanisms of these diseases were not established [232, 233]. Consequently, research on risk factors for disease or indices of health may provide the most immediate information for guiding bighorn sheep management strategies.

## 3. Summary

Over the last century, multiple environmental, host, and agent determinants have been hypothesized as limiting for bighorn sheep populations and/or contributing to outbreaks, and a succession of agents have been investigated. If some credence is given to each report, multiple determinants may actually limit bighorn sheep populations. In addition, some determinants may have multiple effects, such as the potential for high animal densities to favor disease via competition for forage, increased “stress” responses, and increased contact for transmission of infectious agents. These determinants may be present locally or range-wide, and may be altered by climate change or anthropogenic factors. Temporal and spatial variation compounds the challenge of predicting and mitigating disease outbreaks. Furthermore, whether outbreaks are due to completely novel events, and the degree to which endemic agents and environmental conditions favor the occurrence of outbreaks, is unclear. This uncertainty creates challenges for development of management plans and has fostered contention among stakeholders with differing values and competing land use interests.

Field studies of bighorn sheep outbreaks have largely been limited to case reports and cross-sectional studies, and, therefore, have study designs with limited inference [234]. The sporadic nature of outbreaks, limited baseline data, logistic constraints, and other practical concerns present further challenges for developing rigorous study designs of outbreaks. Captive studies of bighorn sheep disease have been employed to circumvent the limitations of field work and address debate between stakeholders on the compatibility of domestic and bighorn sheep under field conditions. However, a clear, invariant relationship between a single agent and field outbreaks has not yet been documented. In part, this could be due to limitations in the available diagnostic assays and practical challenges associated with conducting field work. Therefore, many years of focusing on agents responsible for bighorn sheep respiratory disease have not yielded proven means of predicting and mitigating outbreaks. Consequently, in contrast to a reductionist approach, increased consideration of how host, agent, and environmental determinants may interact under field condition, as well as improved characterization of “healthy” bighorn sheep populations, is needed to expedite development of practical and efficacious management strategies. A more comprehensive approach to disease in bighorn sheep is consistent with domestic animal models of managing “shipping fever” and conventional wildlife management principles [1, 23].

There has been a focus on domestic sheep as a source of infectious agents that are pathogenic to bighorn sheep [5]. This perspective is reasonable because whenever different populations mix, there is the potential for infectious agents to be transmitted from source to naïve populations. This is the basis of regulations restricting interregional movement of animals and plants [235, 236]. However, the actual degree of risk for interspecies transmission of infectious agents, the circumstances where transmission occurs, and practical strategies for minimizing interactions have not been established. In addition, a focus on agent transmission has generally taken precedence over research on competition for forage, behavioral effects, and other factors that might be relevant to interspecies interactions. Given the evidence that interspecies interactions do not invariably result in disease, currently neglected factors may be important for managing these two species in circumstances where: agricultural land is used by bighorn sheep; domestic livestock reside on private land near to bighorn sheep populations; domestic livestock are used for exotic weed control; circumstances where preservation of agricultural enterprises is important for preventing conversion of land to uses that are not compatible with the needs of wildlife; for other reasons. Thus, while protocols for minimizing interspecific transmission of infectious disease from domestic to bighorn sheep have been developed [237], there is a need for further development of practical strategies for minimizing interspecies interactions and conflict between stakeholders.

Recent die-offs of bighorn sheep in several locations suggests that the proximate cause of these events may be a shared environmental determinant. An environmental determinant could favor the development of disease from endogenous or established exotic agents. Drought could be such a determinant if it compromises host health, such as by reducing forage quantity and/or quality. The effect of precipitation quantity and timing on forage may be a useful index of animal health, due to its relationship with bighorn sheep fecundity [51, 98]. Alternatively or concurrently, if these outbreaks were due to the introduction of novel infectious primary pathogens into multiple naive bighorn sheep populations during the same time period, effective strategies for minimizing inter- and intra-population transmission of such agents are needed. Strategies for preventing inter-specific and intraspecific transmission may not need to be agent-specific, and it may be practical to base them on existing wildlife or domestic animal management methods. In addition, prevention of agent transmission and environmental management strategies are not mutually exclusive approaches. Greater attention to host and environmental determinants of disease, as well as validation of methods for limiting epidemics, is likely to complement existing lines of research and result in multiple strategies for predicting and managing outbreaks in bighorn sheep. This is likely to result in programs that are effective in establishing and meeting accepted population management objectives that are based on stakeholder expectations, as well as variation among local and range-wide conditions.

## Figures and Tables

**Table 1:** Causes of direct mortality proposed to limit free-ranging bighorn sheep populations.

Cause of direct mortality	Geographic distribution of determinant	Locations where determinant reported	Selected references
Hunting	Range-wide	Range-wide	[27, 28]
Predation	Range-wide	Alberta, Canada, and Montana	[29, 30]
		Arizona	[31, 32]
		California	[33–36]
		Colorado	[27]
		Montana	[37]
		New Mexico	[38, 39]

**Table 2:** Environmental determinants proposed to limit free-ranging bighorn sheep populations.

Determinant (subcategories)	Geographic distribution of determinant	Locations where determinant reported	Die-off attributed to determinant^1^	Selected references
Adverse environmental conditions	Northern and mountain locations	California	Yes	[28]
		Colorado	No	[42]
		Canadian Rockies, Canada	Yes	[72]
		Wyoming	Yes	[43]
Escape terrain	Range-wide	Arizona	No	[83]
		Baja California Sur, Mexico	No	[84]
		Montana	No	[85]
		New Mexico	No	[44]
Range and migration restriction due to human settlement or activities	Range-wide	Alberta, Canada	No	[86]
		Arizona	No	[87]
		California	Unclear^2^	[28]
		California	No	[88]
		Colorado	No	[27, 89–91]
		Colorado	Yes	[92] (also see [93])
		Montana	No	[94]
Free-water	Primarily arid regions	Desert, general	No	[95]
		Arizona	No	[96, 97]
		California	No	[54, 79]
		California	No	[53]
		Utah	No	[55]

*Food quantity*				
Limited forage and precipitation	Potentially range-wide	Alberta	No	[59]
		Arizona	No	[60, 98, 99]
		California	Yes	[65]
		Colorado	No	[61]
		Montana	Yes	[49]
		Nevada	No	[50]
		New Mexico	No	[44, 62]
		Texas	No	[63, 66]
		Utah	Yes	[51]
Plant community succession	Potentially range-wide	British Columbia, Canada	No	[73]
		California	No	[74, 75]
		Canadian Rockies, Canada	Yes	[72, 76]
		Montana	No	[94]

*Food quality*				
Protein deficiency	Potentially range-wide	Colorado	No	[27]
		Texas	No	[63]
Dietary mineral availability	Range-wide	Alberta, Canada	No	[100]
		Arizona	No	[60, 101]
		British Columbia, Canada	No	[102, 103]
		California	No	[28]
		Colorado	No	[27]
		Utah	No	[104]
		Wyoming	No	[105]
Selenium deficiency	Localized	Wyoming	Yes	[70]

*Competition*				
Competition for forage-domestic species	Range-wide	Alberta, Canada	Yes	[106]
		Arizona	Unclear^2^	[107]
		California	No	[28, 78]
		Colorado	No	[27, 89]
		Nevada	No	[108]
		Oregon	Yes	[109]
		Texas	No	[110]
		Wyoming	Yes	[111]
Competition for forage-bighorn sheep	Potentially range-wide	Colorado	Yes	[112]
		Montana	Yes	[113, 114]
		Tiburon Island, Mexico	No	[3]
		Wyoming	No	[115, 116]
Competition for forage-native ruminant species	Potentially range-wide	Canadian Rockies, Canada	Yes	[72]
		Colorado	No	[27, 89]
		Colorado	Yes	[112]
		Montana	Yes	[117]
		Montana	No	[94]
		Sierra Nevada, California	Unclear^2^	[28]
		Wyoming	No	[118]
		Wyoming	Yes	[111, 113, 119]

*Seasonal*				
Competition for forage-limited winter range (environmental or nonspecific)	Potentially range-wide	Alberta and British Columbia, Canada	Yes	[120, 121]
		Colorado	No	[42]
Competition for forage-limited winter range (domestic livestock grazing)		Colorado	No	[4, 27]
Competition for forage-limited winter range (native ruminant species)		Wyoming	Yes	[122]
Competition for space (social impacts)	Potentially range-wide	Alberta, Canada	No	[59, 80]
		California	No	[79]
		Colorado	Yes	[19]
		Idaho	No	[81]

^1^Mortality considered to be in excess of baseline levels;

^2^Mortality in excess of baseline versus endemic disease status was not clear.

**Table 3:** Infectious agent determinants proposed to limit free-ranging bighorn sheep populations.

Agent category	Infectious agent	Geographic distribution of determinant	Locations where determinant reported	Die-off attributed to determinant^1^	Selected references
Parasite	*Psoroptes* spp.	Range-wide	Arizona	Yes	[143]
			California	Yes	[28]
			Colorado	Yes	[27, 157]
			Montana	Yes	[130]
			Nevada	Yes	[130]
			New Mexico	Yes	[139, 158–161]
			Oregon	Yes	[109, 140]
			Texas	Yes	[110]
			Washington	Yes	[140]
			Wyoming	Yes	[118, 130, 131, 141, 162]
	Lungworm (*Protostrongylus*)	Range-wide in mesic habitats	Alberta, Canada	Yes	[163]
			California	No	[164]
			Colorado	No	[27]
			Colorado	Yes	[146, 165]
			Montana	Yes	[113, 166]
			Montana	No	[148, 167]
			Nevada	No	[168]
			Oregon	No	[149]
			Utah	No	[169]
			Wyoming	Unclear^2^	[118, 122, 170]
	Lungworm (*Muellerius capillaris*)	Localized	Montana	No	[128]
			South Dakota	No	[171]

Bacteria	Pasteurellaceae	Range-wide	Alberta	Yes	[151]
			Arizona	No	[172]
			California	No	[164]
			Colorado	No	[27]
			Colorado	Yes	[19]
			Hells Canyon (Washington, Idaho)	Yes	[18, 152, 173]
			Idaho	No	[174]
			Montana	Yes	[17]
			Montana	No	[128]
			Nevada	No (endemic)	[175]
			Oregon	Yes	[176, 177]
			Wyoming	Yes	[43]
	*Arcanobacterium *(*Corynebacterium*)	Presumed range-wide	Colorado	Yes	[157]
	*pyogenes*		Montana	Yes	[113, 166]
	*Mycoplasma*	Uncertain	Arizona	Yes	[178]
			Hells Canyon	Yes	[18, 179]
	*Chlamydophila *(*Chlamydia*)* Psittaci *	Uncertain	Wyoming	Yes	[180]

Virus	PI3^3^, RSV^4^	Presumed range-wide	British Columbia, Canada	No	[181]
	PI^3^, BRSV^4^	Presumed range-wide	Hell's Canyon	Yes	[18]
	PI3^3^, BRSV^4^, BVD^5^, IBR^6^	Presumed range-wide	Montana	Yes (some populations)	[17]
	PI3^3^, RSV^4^, BVD^5^, IBR^6^, OPP^7^, BT^8^, EHD^9^	Presumed range-wide	California	No	[164, 182, 183]
	PI3^3^, BVD^5^, BT^8^, parvo virus	Presumed range-wide	Colorado and Wyoming	No	[184]
	RSV^4^	Presumed range-wide	Arizona, California, Idaho, Montana, Nevada, New Mexico, Oregon, Utah, Washington	No	[185]
			Colorado	No	[186]
	BRSV^4^, BT^8^, EHD^9^, CE^10^	Presumed range-wide	Arizona	No (EHD and BT isolated from 2 mortalities)	[60, 187]
	BT^8^	Presumed range-wide	Trans-Pecos, Texas	No	[188]
	CE^10^	Presumed range-wide	Alberta, British Columbia, Canada	No (high morbidity, but low mortality)	[189, 190]

^1^Mortality considered to be in excess of baseline levels;

^2^Mortality in excess of baseline versus endemic disease status was not clear;

^3^PI3: parainfluenza-3 virus;

^4^BRSV and RSV: bovine respiratory syncytial virus;

^5^BVD: bovine viral diarrhea virus;

^6^IBR: infectious bovine rhinotracheitis virus;

^7^OPP: ovine progressive pneumonia virus;

^8^BT: bluetongue virus;

^9^EHD: epizootic hemorrhagic disease virus;

^10^CE: contagious ecthyma virus.

## References

[1] Leopold A (1930). *Game Management*.

[2] Gross JE (2001). Evaluating effects of an expanding mountain goat population on native bighorn sheep: a simulation model of competition and disease. *Biological Conservation*.

[3] Colchero F, Medellin RA, Clark JS, Lee R, Katul GG (2009). Predicting population survival under future climate change: density dependence, drought and extraction in an insular bighorn sheep. *Journal of Animal Ecology*.

[4] Spraker TR, Adrian WJ (1990). Problems with “multiple land use” dealing with bighorn sheep and domestic livestock. *Biennial Symposium of the Northern Wild Sheep and Goat Council*.

[5] United States Geologic Survey/Bureau of Reclamation Office (2006). Payette National Forest Science Panel Discussion on risk for disease transmission analysis between bighorn and domestic sheep.

[6] Toweill DE, Geist V (1999). *Return of Royalty: Wild Sheep of North America*.

[7] Buechner HK (1960). The bighorn sheep in the United States, its past, present, and future. *Wildlife Monographs*.

[8] Gross JE, Singer FJ, Moses ME, Krausman PR (2000). Effects of disease, dispersal, and area on bighorn sheep restoration. *Restoration Ecology*.

[9] Council for Agricultural Science and Technology (CAST) (2008). *Pasteurellosis Transmission Risks between Domestic and Wild Sheep*.

[10] Hodo C (2010). Pnuemonia kills bighorn sheep. *SCWDS Briefs*.

[11] Pillmore RE (1958). Life cycle of the lungworm genus *Protostrongylus* in Colorado. *Journal of the Colorado-Wyoming Academy of Science*.

[12] Forrester DJ, Davis JW, Anderson RC (1971). Bighorn sheep lungworm-pneumonia complex. *Parasitic Diseases of Wild Mammals*.

[13] Onderka DK, Rawluk SA, Wishart WD (1988). Susceptibility of Rocky Mountain bighorn sheep and domestic sheep to pneumonia induced by bighorn and domestic livestock strains of *Pasteurella haemolytica*. *Canadian Journal of Veterinary Research*.

[14] Foreyt WJ, Snipes KP, Kasten RW (1994). Fatal pneumonia following inoculation of healthy bighorn sheep with *Pasteurella haemolytica* from healthy domestic sheep. *Journal of Wildlife Diseases*.

[15] Dassanayake RP, Shanthalingam S, Herndon CN (2009). *Mannheimia haemolytica* serotype A1 exhibits differential pathogenicity in two related species, *Ovis* canadensis and *Ovis aries*. *Veterinary Microbiology*.

[16] Lawrence PK, Shanthalingam S, Dassanayake RP (2010). Transmission of *Mannheimia haemolytica* from domestic sheep (*Ovis aries*) to bighorn sheep (*Ovis canadensis*): unequivocal demonstration with green fluorescent protein-tagged organisms. *Journal of Wildlife Diseases*.

[17] Aune K, Anderson N, Worley DE, Stackhouse L, Henderson J, Daniel J (1998). A comparison of population and health histories among seven Montana bighorn sheep populations. *Biennial Symposium of the Northern Wild Sheep and Goat Council*.

[18] Rudolph KM, Hunter DL, Rimler RB (2007). Microorganisms associated with a pneumonic epizootic in Rocky Mountain bighorn sheep (*Ovis canadensis* canadensis). *Journal of Zoo and Wildlife Medicine*.

[19] George JL, Martin DJ, Lukacs PM, Miller MW (2008). Epidemic pasteurellosis in a bighorn sheep population coinciding with the appearance of a domestic sheep. *Journal of Wildlife Diseases*.

[20] Andryk TA, Irby LR (1986). Population characteristics and habitat use by mountain sheep prior to a pneumonia die-off. *Biennial Symposium of the Northern Wild Sheep and Goat Council*.

[21] Wobesor GA (1994). *Investigation and Management of Disease in Wild Animals*.

[22] Schwabe CW, Riemann HP, Franti CE (1977). *Epidemiology in Veterinary Practice*.

[23] Yates WDG (1982). A review of infectious bovine rhinotracheitis, shipping fever pneumonia and viral-bacterial synergism in respiratory disease of cattle. *Canadian Journal of Comparative Medicine*.

[24] Czuprynski CJ, Leite F, Sylte M (2004). Complexities of the pathogenesis of *Mannheimia haemolytica* and *Haemophilus somnus* infections: challenges and potential opportunities for prevention?. *Animal Health Research Reviews*.

[25] Zecchinon L, Fett T, Desmecht D (2005). How *Mannheimia haemolytica* defeats host defence through a kiss of death mechanism. *Veterinary Research*.

[26] Dabo SM, Taylor JD, Confer AW (2007). *Pasteurella* multocida and bovine respiratory disease. *Animal Health Research Reviews*.

[27] Packard FM (1946). An ecological study of the bighorn sheep in Rocky Mountain National Park. *Journal of Mammalogy*.

[28] Jones FL (1950). A survey of the Sierra Nevada bighorn. *Sierra Club Bulletin*.

[29] Ross PI, Jalkotzy MG, FestaBianchet M (1997). Cougar predation on bighorn sheep in southwestern Alberta during winter. *Canadian Journal of Zoology-Revue Canadienne de Zoologie*.

[30] Festa-Bianchet M, Coulson T, Gaillard JM, Hogg JT, Pelletier F (2006). Stochastic predation events and population persistence in bighorn sheep. *Proceedings of the Royal Society B*.

[31] Kamler JF, Lee RM, DeVos JC, Ballard WB, Whitlaw HA (2002). Survival and cougar predation of translocated bighorn sheep in Arizona. *The Journal of Wildlife Management*.

[32] McKinney T, Devos JC, Ballard WB, Boe SR (2006). Mountain lion predation of translocated desert bighorn sheep in Arizona. *Wildlife Society Bulletin*.

[33] Schaefer RJ, Torres SG, Bleich VC (2000). Survivorship and cause-specific mortality in sympatric populations of mountain sheep and mule deer. *California Fish and Game*.

[34] Hayes CL, Rubin ES, Jorgensen MC, Botta RA, Boyce WM (2000). Mountain lion predation of bighorn sheep in the Peninsular Ranges, California. *The Journal of Wildlife Management*.

[35] Ernest HB, Rubin ES, Boyce WM (2002). Fecal DNA analysis and risk assessment of mountain lion predation of bighorn sheep. *The Journal of Wildlife Management*.

[36] Wehausen JD (1996). Effects of mountain lion predation on bighorn sheep in the Sierra Nevada and Granite Mountains of California. *Wildlife Society Bulletin*.

[37] Legg K, Irby LR, Lempke T (1996). An analysis of potential factors responsible for the decline in bighorns in the Tom Miner Basin. *Biennial Symposium of the Northern Wild Sheep and Goat Council*.

[38] Rominger EM, Whitlaw HA, Weybright DL, Dunn WC, Ballard WB (2004). The influence of mountain lion predation on bighorn sheep translocations. *The Journal of Wildlife Management*.

[39] Mooring MS, Fitzpatrick TA, Nishihira TT, Reisig DD (2004). Vigilance, predation risk, and the Allee effect in desert bighorn sheep. *The Journal of Wildlife Management*.

[40] Chamberlin TC (1965). The method of multiple working hypotheses. *Science*.

[41] Cassirer EF, Sinclair ARE (2007). Dynamics of pneumonia in a bighorn sheep metapopulation. *The Journal of Wildlife Management*.

[42] Goodson NJ, Stevens DR, Bailey JA (1991). Effects of snow on foraging ecology and nutrition of bighorn sheep. *The Journal of Wildlife Management*.

[43] Ryder TJ, Williams ES, Mills KW, Bowles KH, Thorne ET (1992). Effect of pneumonia on population size and lamb recruitment in Whisky Mountain bighorn sheep. *Biennial Symposium of the Northern Wild Sheep and Goat Council*.

[44] Dunn WC (1993). *Evaluation of Rocky Mountain Bighorn Sheep Habitat in New Mexico*.

[45] Risenhoover KL, Bailey JA (1985). Foraging ecology of mountain sheep: implications for habitat management. *The Journal of Wildlife Management*.

[46] Festa-Bianchet M (1988). Seasonal range selection in bighorn sheep: conflicts between forage quality, forage quantity, and predator avoidance. *Oecologia*.

[47] Geist V (1971). *Mountain Sheep, a Study in Behavior and Evolution*.

[48] Risenhoover KL, Bailey JA, Wakelyn LA (1988). In My Opinion ... Assessing the Rocky Mountain bighorn sheep management problem. *Wildlife Society Bulletin*.

[49] Enk TA, Picton HD, Williams JS (2001). Factors limiting a bighorn sheep population in Montana following a dieoff. *Northwest Science*.

[50] Douglas CL, Leslie DM (1986). Influence of weather and density on lamb survival of desert mountain sheep. *The Journal of Wildlife Management*.

[51] Douglas CL (2001). Weather, disease, and bighorn lamb survival during 23 years in Canyonlands National Park. *Wildlife Society Bulletin*.

[52] Merwin DS, Brundige GC (2000). An unusual contagious ecthyma outbreak in Rock Mountain bighorn sheep. *Biennial Symposium of the Northern Wild Sheep and Goat Council*.

[53] Turner JC, Douglas CL, Hallum CR, Krausman PR, Ramey RR (2004). Determination of critical habitat for the endangered Nelson’s bighorn sheep in southern California. *Wildlife Society Bulletin*.

[54] Longshore KM, Lowrey C, Thompson DB (2009). Compensating for diminishing natural water: predicting the impacts of water development on summer habitat of desert bighorn sheep. *Journal of Arid Environments*.

[55] Whiting JC, Bowyer RT, Flinders JT (2009). Annual use of water sources by reintroduced Rocky Mountain bighorn sheep *Ovis canadensis* canadensis: effects of season and drought. *Acta Theriologica*.

[56] Rosenstock SS, Hervert JJ, Bleich VC, Krausman PR (2001). Muddying the water with poor science: a reply to Broyles and Cutler. *Wildlife Society Bulletin*.

[57] Broyles B, Cutler TL (2001). Reply to Rosenstock et al. (2001) regarding effects of water on desert bighorn sheep at Cabeza Prieta National Wildlife Refuge, Arizona. *Wildlife Society Bulletin*.

[58] Cain JW, Krausman PR, Morgart JR, Jansen BD, Pepper MP (2008). Responses of desert bighorn sheep to removal of water sources. *Wildlife Monographs*.

[59] Portier C, Festa-Bianchet M, Gaillard JM, Jorgenson JT, Yoccoz NG (1998). Effects of density and weather on survival of bighorn sheep lambs (*Ovis canadensis*). *Journal of Zoology*.

[60] McKinney T, Smith TW, DeVos JC (2006). Evaluation of factors potentially influencing a desert bighorn sheep population. *Wildlife Monographs*.

[61] Goodson NJ, Stevens DR, Bailey JA (1991). Winter-spring foraging ecology and nutrition of bighorn sheep on montane ranges. *The Journal of Wildlife Management*.

[62] Bender LC, Weisenberger ME (2005). Precipitation, density, and population dynamics of desert bighorn sheep on San Andres National Wildlife Refuge, New Mexico. *Wildlife Society Bulletin*.

[63] Deyoung RW, Hellgren EC, Fulbright TE, Robbins WF, Humphreys ID, Krausman PR (2000). Modelling nutritional carrying capacity for translocated desert bighorn sheep in Western Texas. *Restoration Ecology*.

[64] Rominger EM, Goldstein EJ, Evans MA (2008). Biological and statistical errors make inferences circumspect: response to Bender and Weisenberger. *The Journal of Wildlife Management*.

[65] Wehausen JD, Vernon CB, Blong B, Russi TL (1987). Recruitment dynamics in a Southern California mountain sheep population. *The Journal of Wildlife Management*.

[66] Brewer CE, Harveson LA (2007). Diets of bighorn sheep in the Chihuahuan Desert, Texas. *Southwestern Naturalist*.

[67] Ganskopp D, Bohnert D (2001). Nutritional dynamics of 7 northern great basin grasses. *Journal of Range Management*.

[68] Nordheim-Viken H, Volden H (2009). Effect of maturity stage, nitrogen fertilization and seasonal variation on ruminal degradation characteristics of neutral detergent fibre in timothy (*Phleum pratense* L.). *Animal Feed Science and Technology*.

[69] Cook JG, Johnson BK, Cook RC, Wisdom MJTE (2005). Nutrition and parturition date effects on elk: potential implications for research and management. *The Starkey Project: A Synthesis of Long-Term Studies of Elk and Mule Deer*.

[70] Hnilicka P, Mioncznski J, Mincher BJ (2002). Bighorn sheep lamb survival, trace minerals, rainfall, and air pollution: are there any connections?. *Biennial Symposium of the Northern Wild Sheep and Goat Council*.

[71] Keating KA (1990). Bone chewing by Rocky-Mountain Bighorn sheep. *Great Basin Naturalist*.

[72] Stelfox JG (1971). Bighorn sheep in the Canadian Rockies: a history 1800–1970. *Canadian Field-Naturalist*.

[73] Dibb AD, Quinn MS (2008). Response of bighorn sheep to restoration of Winter Range: revisited. *Biennial Symposium of the Northern Wild Sheep and Goat Council*.

[74] Holl SA, Bleich VC, Torres SG (2004). Population dynamics of bighorn sheep in the San Gabriel Mountains, California, 1967–2002. *Wildlife Society Bulletin*.

[75] Bleich VC, Johnson HE, Holl SA, Konde L, Torres SG, Krausman PR (2008). Fire history in a chaparral ecosystem: implications for conservation of a native ungulate. *Rangeland Ecology and Management*.

[76] Bentz JA, Woodard PM (1988). Vegetation characteristics and bighorn sheep use on burned and unburned areas in Alberta. *Wildlife Society Bulletin*.

[77] Epps CW, McCullough DR, Wehausen JD, Bleich VC, Rechel JL (2004). Effects of climate change on population persistence of Desert-Dwelling Mountain sheep in California. *Conservation Biology*.

[78] Wright GM, Dixon JS, Thompson BH (1933). *Fauna of the National Parks of the Unites States*.

[79] Ostermann-Kelm S, Atwill ER, Rubin ES, Jorgensen MC, Boyce WM (2008). Interactions between feral horses and desert bighorn sheep at water. *Journal of Mammalogy*.

[80] Festa-Bianchet M, Jorgenson JT (1998). Selfish mothers: reproductive expenditure and resource availability in bighorn ewes. *Behavioral Ecology*.

[81] Bissonette JA, Steinkamp MJ (1996). Bighorn sheep response to ephemeral habitat fragmentation by cattle. *Great Basin Naturalist*.

[82] Enk TA (1999). *Population dynamics of bighorn sheep on the Beartooth Wildlife Management Area, Montana*.

[83] McKinney T, Boe SR, DeVos JC (2003). GIS-based evaluation of escape terrain and desert bighorn sheep populations in Arizona. *Wildlife Society Bulletin*.

[84] Alvarez-Cárdenas S, Guerrero-Cárdenas I, Díaz S, Galina-Tessaro P, Gallina S (2001). The variables of physical habitat selection by the desert bighorn sheep (*Ovis canadensis* weemsi) in the Sierra del Mechudo, Baja California Sur, México. *Journal of Arid Environments*.

[85] Beyer AC (2008). *Habitat comparisons of historically stable and less stable bighorn sheep populations*.

[86] Horejsi B (1976). Some thoughts and observations on harassment and bighorn sheep. *Proceedings of the Biennial Symposium of the Northern Wild Sheep and Goat Council*.

[87] Stockwell CA, Bateman GC, Berger J (1991). Conflicts in national parks: a case study of helicopters and bighorn sheep time budgets at the grand canyon. *Biological Conservation*.

[88] Bleich VC, Wehausen JD, Holl SA (1990). Desert-dwelling mountain sheep—conservation Implications of a Naturally Fragmented Distribution. *Conservation Biology*.

[89] Bear GD, Jones GW (1973). History and distribution of bighorn sheep in Colorado. *Colorado Division of Wildlife Federal Aid Project W-41-R-22, Job and Final Report*.

[90] Pillmore RE (1958). Problems of lungworm infection in wild sheep. *Desert Bighorn Council Transactions*.

[91] Keller BJ, Bender LC (2007). Bighorn sheep response to road-related disturbances in Rocky Mountain National Park, Colorado. *The Journal of Wildlife Management*.

[92] Bailey JA (1986). The increase and die-off of Waterton Canyon bighorn sheep: biology, management, and dismanagement. *Biennial Symposium of the Northern Wild Sheep and Goat Council*.

[93] Spraker TR, Hibler CP, Schoonveld GG, Adney WS (1984). Pathologic changes and microorganisms found in bighorn sheep during a stress-related die-off. *Journal of Wildlife Diseases*.

[94] Berwick SH (1968). *Observations on the decline of the Rock Creek, Montana population of bighorn sheep*.

[95] Broyles B (1995). Desert wildlife water developments: questioning use in the Southwest. *Wildlife Society Bulletin*.

[96] Krausman PR, Etchberger RC (1995). Response of desert ungulates to a water project in Arizona. *The Journal of Wildlife Management*.

[97] Broyles B, Cutler TL (2000). Effect of surface water on desert bighorn sheep in the Cabeza Prieta National Wildlife Refuge, southwestern Arizona. *Wildlife Society Bulletin*.

[98] McKinney T, Smith TW, Hanna JD (2001). Precipitation and desert bighorn sheep in the Mazatzal Mountains, Arizona. *Southwestern Naturalist*.

[99] McKinney T, Smith TW (2007). Diets of adults and lambs of desert bighorn sheep during years of varying rainfall in Central Arizona. *Southwestern Naturalist*.

[100] Skipworth JP (1974). Ingestion of Grit by Bighorn sheep. *The Journal of Wildlife Management*.

[101] Warrick G, Krausman PR (1986). Bone-Chewing by desert Bighorn sheep. *Southwestern Naturalist*.

[102] Hebert DM (1972). Forage and serum phosphorus values for Bighorn sheep. *Journal of Range Management*.

[103] Schuerholz G (1984). Annual ecological cycles of Rocky Mountain bighorn sheep populations with high elevation winter ranges in the east Kootenays. *Biennial Symposium of the Northern Wild Sheep and Goat Council*.

[104] Mahon CL (1969). Mineral deficiencies in desert bighorns and domestic livestock in San Juan County. *Desert Bighorn Council Transactions*.

[105] Mincher BJ, Ball RD, Houghton TP, Mionczynski J, Hnilicka PA (2008). Some aspects of geophagia in Wyoming bighorn sheep (*Ovis canadensis*). *European Journal of Wildlife Research*.

[106] Festa-Bianchet M (1988). A pneumonia epizootic in bighorn sheep with comments on preventative management. *Biennial Symposium of the Northern Wild Sheep and Goat Council*.

[107] Gallizoli S (1977). Overgrazing on desert bighorn ranges. *Desert Bighorn Council Transactions*.

[108] McQuivey RP (1978). *The Desert Bighorn Sheep of Nevada*.

[109] Bailey V (1936). Mammals of oregon: *Ovis canadensis* canadensis Shaw. *North American Fauna*.

[110] Davis WB, Taylor WP (1939). The bighorn sheep of Texas. *Journal of Mammalogy*.

[111] Honess RF, Frost NM (1942). *A Wyoming Bighorn Sheep Study*.

[112] Feuerstein V, Schmidt RL, Hibler CP, Rutherford WH (1980). Bighorn sheep mortality in the Taylor River-Almont Triangle area, 1978–1979: a case study. *Special Report*.

[113] Marsh H (1938). Pneumonia in Rocky Mountain bighorn sheep. *Journal of Mammalogy*.

[114] Semmens WJ (1996). *Seasonal movements and habitat use of the Highlands/Pioneer Mountains bighorn sheep herd of southwest Montana*.

[115] Thorne ET, Butler G, Varcalli T, Becker K, Hayden-Wing S (1979). The status, mortality and response to management of the bighorn sheep of Whisky Mountain. *Wildlife Technical Report Number 7*.

[116] Cook JG (1990). *Habitat, nutrition, and population ecology of two transplanted bighorn sheep populations in southcentral Wyoming*.

[117] Couey FM (1955). Montana bighorn sheep. *Proceedings of the Annual Conference of the Western Association of State Game and Fish Commissionser*.

[118] Mills HB (1937). A preliminary study of the bighorn of Yellowstone National Park. *Journal of Mammalogy*.

[119] McCann LJ (1956). Ecology of the mountain sheep. *The American Midland Naturalist*.

[120] Demarchi RA, Demarchi DA (1967). Status of the Rocky Mountain bighorn. *Wildlife Review*.

[121] Stelfox JG (1974). Range ecology of bighorn sheep in relation to self-regulation theories. *Biennial Symposium of the Northern Wild Sheep Council*.

[122] Honess RF, Winters K (1956). What about the bighorn. *Wyoming Wildlife*.

[123] Boyce WM, Hedrick PW, Muggli-Cockett NE, Kalinowski S, Penedo MCT, Ramey RR (1997). Genetic variation of major histocompatibility complex and microsatellite loci: a comparison in bighorn sheep. *Genetics*.

[124] Gutierrez-Espeleta GA, Hedrick PW, Kalinowski ST, Garrigan D, Boyce WM (2001). Is the decline of desert bighorn sheep from infectious disease the result of low MHC variation?. *Heredity*.

[125] Goldstein EJ, Millspaugh JJ, Washburn BE, Brundige GC, Raedeke KJ (2005). Relationships among fecal lungworm loads, fecal glucocorticoid metabolites, and lamb recruitment in free-ranging Rocky Mountain bighorn sheep. *Journal of Wildlife Diseases*.

[126] Malmberg JL, Nordeen T, Butterfield C (2008). The effects of disease, stress, and distribution on bighorn sheep restoration in nebraska. *Biennial Symposium of the Northern Wild Sheep and Goat Council*.

[127] Barker IK, Beveridge I, Bradley AJ, Lee AK (1978). Observations on spontaneous stress-related mortality among males of the dasyurid marsupial *Antechinus stuartii*. *Australian Journal of Zoology*.

[128] Miller DS, Weiser GC, Aune K (2011). Shared bacterial and viral respiratory agents in bighorn (*Ovis canadensis*) and domestic sheep (*Ovis aries*) in Montana. *Veterinary Medicine International*.

[129] Thorne ET, Kingston N, Jolley WR, Bergstrom RC (1982). *Diseases of Wildlife in Wyoming*.

[130] Hornaday WT (1901). Notes on the mountain sheep of North America with a description of a new species. *New York Zoological Society Annual Report*.

[131] Baillie-Grohman WA, Baillie-Grohman WA (1902). Camps on the trail of the bighorn. *Camps in the Rockies*.

[132] Grinnell GB, Grinnell GB (1904). The mountain sheep and its range. *American Big Game in Its Haunts*.

[133] Wright FC, Riner JC, Fisher WF (1984). Comparison of lengths of outer opisthosomal setae of male psoroptic mites collected from various hosts. *Journal of Parasitology*.

[134] Boyce W, Elliott L, Clark R, Jessup D (1990). Morphometric analysis of *Psoroptes* spp. mites from bighorn sheep, mule deer, cattle, and rabbits. *Journal of Parasitology*.

[135] Boyce WM, Brown RN (1991). Antigenic characterization of *Psoroptes* spp. (Acari: Psoroptidae) mites from different hosts. *Journal of Parasitology*.

[136] Graham OH, Hourrigan JL (1977). Eradication programs for the arthropod parasites of livestock. *Journal of Medical Entomology*.

[137] Pugh DG (2002). *Sheep and Goat Medicine*.

[138] van den Broek AHM, Huntley JF, Machell J, Taylor MA, Miller HRP (2003). Temporal pattern of isotype-specific antibody responses in primary and challenge infestations of sheep with *Psoroptes ovis*—the sheep scab mite. *Veterinary Parasitology*.

[139] Sandoval AV (1980). Management of a psoroptic scabies epizootic in bighorn sheep (*Ovis canadensis* mexicana) in New Mexico. *Desert Bighorn Council Transactions*.

[140] Foreyt W, Coggins V, Fowler P (1990). Psoroptic scabies in bighorn sheep in Washington and Oregon. *Biennial Symposium of the Northern Wild Sheep and Goat Council*.

[141] Muschenheim AL, Thorne ET, Williams ES, Anderson SH, Wright FC (1990). Psoroptic scabies in Rocky Mountain bighorn sheep (*Ovis canadensis* canadensis) from Wyoming. *Journal of Wildlife Diseases*.

[142] Boyce WM, Jessup DA, Clark RK (1991). Serodiagnostic antibody responses to *Psoroptes* sp. infestations in bighorn sheep. *Journal of Wildlife Diseases*.

[143] Welsh GW, Bunch TD (1983). Psoroptic scabies in desert bighorn sheep (*Ovis canadensis* nelsoni) from northwestern Arizona. *Journal of Wildlife Diseases*.

[144] Russo JP (1956). *The Desert Bighorn Sheep in Arizona*.

[145] Allen RW (1971). Present status of lungworm and tapeworm in desert bighorn sheep. *Desert Bighorn Council Transactions*.

[146] Spraker TR, Hibler CP (1977). Summer lamb mortality of Rocky Mountain bighorn sheep. *Desert Bighorn Council Transactions*.

[147] Spraker TR (1977). Fibrinous pneumonia of bighorn sheep. *Desert Bighorn Council Transactions*.

[148] Forrester DJ, Littell RC (1976). Influence of rainfall on lungworm infections in bighorn sheep. *Journal of Wildlife Diseases*.

[149] Kistner TP, Matlock SM, Wyse D, Mason GE (1977). Helminth parasites of bighorn sheep in Oregon. *Journal of Wildlife Diseases*.

[150] Pillmore RE (1961). *Investigation of Disease and Parasites Affecting Game Animals: Study of the Lung Nematodes of Bighorn Sheep*.

[151] Onderka DK, Wishart WD (1984). A major sheep die-off from pneumonia in southern Alberta. *Biennial Symposium of the Northern Wild Sheep and Goat Council*.

[152] Cassirer EF, Oldenburg LE, Coggins V (1996). Overview and preliminary analysis of a bighorn sheep dieoff, Hells Canyon 1995-96. *Biennial Symposium of the Northern Wild Sheep and Goat Council*.

[153] Foreyt WJ, Jessup DA (1982). Fatal pneumonia of bighorn sheep following association with domestic sheep. *Journal of Wildlife Diseases*.

[154] Samson J, Holmes JC, Jorgenson JT, Wishart WD (1987). Experimental infections of free-ranging Rocky Mountain bighorn sheep with lungworms (*Protostrongylus* spp.; Nematoda: Protostrongylidae). *Journal of Wildlife Diseases*.

[155] Forrester DJ, Senger CM (1964). A survey of lungworm infection in bighorn sheep of Montana. *The Journal of Wildlife Management*.

[156] Miller MW, Vayhinger JE, Bowden DC (2000). Drug treatment for lungworm in bighorn sheep: reevaluation of a 20-year-old management prescription. *The Journal of Wildlife Management*.

[157] Potts MK (1938). Observations on diseases of bighorn in Rocky Mountain National Park. *Transactions of the North American Wildlife Conference*.

[158] Meleney WP, Wright FC, Guillot FS (1980). Identification and control of psoroptic scabies in bighorn sheep (*Ovis canadensis* mexicana). *Proceedings of the Annual Meeting of the United States Animal Health Association*.

[159] Kinzer HG, Houghton WE, Reeves JM (1983). *Psoroptes ovis* research with bighorn sheep in New Mexico. *Desert Bighorn Council Transactions*.

[160] Boyce WM, Clark RK, Jessup DA (1990). Recent advances in the diagnosis and treatment of psoroptic scabies in bighorn sheep. *Biennial Symposium of the Northern Wild Sheep and Goat Council*.

[161] Boyce WM, Weisenberger ME (2005). The rise and fall of psoroptic scabies in bighorn sheep in the San Andres Mountains, New Mexico. *Journal of Wildlife Diseases*.

[162] Rush WM (1928). Diseases in mountain sheep. *Outdoor Life*.

[163] Wishart WD, Jorgenson J, Hilton M (1980). A minor die-off of bighorns from pneumonia in southern Alberta. *Biennial Symposium of the Northern Wild Sheep and Goat Council*.

[164] Clark RK, Jessup DA, Kock MD, Weaver RA (1985). Survey of desert bighorn sheep in California for exposure to selected infectious diseases. *Journal of the American Veterinary Medical Association*.

[165] Woodward T, Hibler CP, Rutherford WH (1972). Bighorn lamb mortality investigations in Colorado. *Transactions of the Northern Wild Sheep Council*.

[166] Evans HF (1937). Bighorn at many glacier. *Glacial Drift*.

[167] Arnett EB, Irby LR, Cook JG (1993). Sex- and age-specific lungworm infection in Rocky Mountain bighorn sheep during winter. *Journal of Wildlife Diseases*.

[168] Allen RW (1964.). Additional notes on parasites of bighorn sheep on the Desert Game Range, Nevada. *Desert Bighorn Council Transactions*.

[169] Rogerson JD, Fairbanks WS, Cornicelli L (2008). Ecology of gastropod and bighorn sheep hosts of lungworms on isolated, semiarid mountain ranges in Utah, USA. *Journal of Wildlife Diseases*.

[170] Honess RF (1942). *Lungworms of Domestic Sheep and Bighorn Sheep in Wyoming*.

[171] Pybus MJ, Shave H (1984). Muellerius capillaris (Mueller, 1889) (Nematoda: Protostrongylidae): an unusual finding in Rocky Mountain bighorn sheep (*Ovis canadensis* canadensis Shaw) in South Dakota. *Journal of Wildlife Diseases*.

[172] Jaworski MD, Hunter DL, Ward ACS (1998). Biovariants of isolates of *Pasteurella* from domestic and wild ruminants. *Journal of Veterinary Diagnostic Investigation*.

[173] WeIser GC, DeLong WJ, Paz JL, Shafii B, Price WJ, Ward ACS (2003). Characterization of *Pasteurella* multocida associated with pneumonia in bighorn sheep. *Journal of Wildlife Diseases*.

[174] Dunbar MR, Ward AC, Power G (1990). Isolation of *Pasteurella haemolytica* from tonsillar biopsies of Rocky Mountain bighorn sheep. *Journal of Wildlife Diseases*.

[175] Taylor RE (1976). Mortality of Nevada bighorn sheep from pneumonia. *Desert Bighorn Council Transactions*.

[176] Coggins V (1988). The Lostine Rocky Mountain bighorn sheep die-off and domestic sheep. *Biennial Symposium of the Northern Wild Sheep and Goat Council*.

[177] Coggins V, Matthews PE (1992). Lamb survival and herd status of the Lostine bighorn herd following a *Pasteurella* die-off. *Biennial Symposium of the Northern Wild Sheep and Goat Council*.

[178] Jansen BD, Heffelfinger JR, Noon TH, Krausman PR, deVos JC (2006). Infectious keratoconjunctivitis in Bighorn sheep, Silver Bell Mountains, Arizona, USA. *Journal of Wildlife Diseases*.

[179] Besser TE, Cassirer EF, Potter KA (2008). Association of Mycoplasma ovipneumoniae infection with population-limiting respiratory disease in free-ranging Rocky Mountain bighorn sheep (*Ovis canadensis* canadensis). *Journal of Clinical Microbiology*.

[180] Meagher M, Quinn WJ, Stackhouse L (1992). Chlamydial-caused infectious keratoconjunctivitis in bighorn sheep of Yellowstone National Park. *Journal of Wildlife Diseases*.

[181] Schwantje H (1986). A comparative study of bighorn sheep herds in southeastern British Columbia. *Biennial Symposium of the Northern Wild Sheep and Goat Council*.

[182] Clark RK, Boyce WM, Jessup DA, Elliott LF (1993). Survey of pathogen exposure among population clusters of bighorn sheep (*Ovis canadensis*) in California. *Journal of Zoo and Wildlife Medicine*.

[183] Clark RK, Whetstone CA, Castro AE, Jorgensen MM, Jensen JF, Jessup DA (1993). Restriction endonuclease analysis of herpesviruses isolated from two peninsular bighorn sheep (*Ovis canadensis* cremnobates). *Journal of Wildlife Diseases*.

[184] Parks JB, England JJ (1974). A serological survey for selected viral infections of Rocky Mountain bighorn sheep. *Journal of Wildlife Diseases*.

[185] Dunbar MR, Jessup DA, Evermann JF, Foreyt WJ (1985). Seroprevalence of respiratory syncytial virus in free-ranging bighorn sheep. *Journal of the American Veterinary Medical Association*.

[186] Spraker TR, Collins JK, Adrian WJ, Olterman JH (1986). Isolation and serologic evidence of a respiratory syncytial virus in bighorn sheep from Colorado. *Journal of Wildlife Diseases*.

[187] Noon TH, Wesche SL, Cagle D (2002). Hemorrhagic disease in bighorn sheep in Arizona. *Journal of Wildlife Diseases*.

[188] Robinson RM, Hailey TL, Livingston CW, Thomas JW (1967). Bluetongue in desert Bighorn sheep. *The Journal of Wildlife Management*.

[189] Samuel WM, Chalmers GA, Stelfox JG, Loewen A, Thomsen JJ (1975). Contagious ecthyma in bighorn sheep and mountain goat in western Canada. *Journal of Wildlife Diseases*.

[190] Blood DA (1971). Contagious ecthyma in Rocky-Mountain Bighorn sheep. *The Journal of Wildlife Management*.

[191] Ezenwa VO, Hines AM, Archie EA, Hoberg EP, Asmundsson IM, Hogg JT (2010). Muellerius capillaris dominates the lungworm community of bighorn sheep at the national bison range, Montana. *Journal of Wildlife Diseases*.

[192] Hoberg EP, Brooks DR (2008). A macroevolutionary mosaic: episodic host-switching, geographical colonization and diversification in complex host-parasite systems. *Journal of Biogeography*.

[193] Huby-Chilton F, Gajadhar AA, Mansfield K, Foreyt WJ, Chilton NB (2006). Bighorn sheep, a new host record for *Parelaphostrongylus odocoilei* (Nematoda: Protostrongylidae). *Journal of Wildlife Diseases*.

[194] Jenkins EJ, Veitch AM, Kutz SJ (2007). Protostrongylid parasites and pneumonia in captive and wild thinhorn sheep (*Ovis dalli*). *Journal of Wildlife Diseases*.

[195] Hibler CP, Metzger CJ, Spraker TR, Lange RE (1974). Further observations on *Protostrongylus* sp. infection by transplacental transmission in bighorn sheep. *Journal of Wildlife Diseases*.

[196] Samuel WM, Gray JB (1982). Evaluation of the Baermann technic for recovery of lungworm (Nematoda, Protostrongylidae) larvae from wild ruminants. *Biennial Symposium of the Northern Wild Sheep and Goat Council*.

[197] Beane RD, Hobbs NT (1983). The Baermann technique for estimating *Protostrongylus* infection in bighorn sheep: effect of laboratory procedures. *Journal of Wildlife Diseases*.

[198] Uhazy LS, Holmes JC, Stelfox JG (1973). Lungworms in the Rocky Mountain bighorn sheep of Western Canada. *Canadian Journal of Zoology*.

[199] Forrester SG, Lankester MW (1997). Extracting protostrongylid nematode larvae from ungulate feces. *Journal of Wildlife Diseases*.

[200] Kutz SJ, Asmundsson I, Hoberg EP (2007). Serendipitous discovery of a novel protostrongylid (Nematoda: Metastrongyloidea) in caribou, muskoxen, and moose from high latitudes of North America based on DNA sequence comparisons. *Canadian Journal of Zoology-Revue Canadienne de Zoologie*.

[201] Paul SR, Bunch TD (1978). Chronic frontal sinusitis and osteolysis in desert bighorn sheep. *Journal of the American Veterinary Medical Association*.

[202] Uhazy LS, Mahrt JL, Holmes JC (1971). Coccidia of Rocky Mountain bighorn sheep in Western Canada. *Canadian Journal of Zoology*.

[203] Worley DE, Seesee F (1992). Gastrointestinal parasites of bighorn sheep in western Montana and their relationship to herd health. *Colorado Division of Wildlife Federal Aid Project W-41-r-22, Job and Final Report*.

[204] de la Fuente J, Atkinson MW, Hogg JT (2006). Genetic characterization of Anaplasma ovis strains from Bighorn sheep in Montana. *Journal of Wildlife Diseases*.

[205] Kutz SJ, Hoberg EP, Polley L, Jenkins EJ (2005). Global warming is changing the dynamics of Arctic host-parasite systems. *Proceedings of the Royal Society B*.

[206] Jenkins EJ, Veitch AM, Kutz SJ, Hoberg EP, Polley L (2006). Climate change and the epidemiology of protostrongylid nematodes in northern ecosystems: *Parelaphostrongylus odocoilei* and *Protostrongylus* stilesi in Dall’s sheep (Ovis d. dalli). *Parasitology*.

[207] Hoberg EP, Polley L, Jenkins EJ, Kutz SJ (2008). Pathogens of domestic and free-ranging ungulates: global climate change in temperate to boreal latitudes across North America. *OIE Revue Scientifique et Technique*.

[208] Brogden KA, Lehmkuhl HD, Cutlip RC (1998). *Pasteurella haemolytica* complicated respiratory infections in sheep and goats. *Veterinary Research*.

[209] Ackermann MR, Brogden KA (2000). Response of the ruminant respiratory tract to *Mannheimia (Pasteurella) haemolytica*. *Microbes and Infection*.

[210] Kehrenberg C, Schulze-Tanzil G, Martel JL, Chaslus-Dancla E, Schwarz S (2001). Antimicrobial resistance in *Pasteurella* and *Mannheimia*: epidemiology and genetic basis. *Veterinary Research*.

[211] Miller MW, Wolfe LL (2011). Pasteurellaceae from Colorado Bighorn Sheep Herds. *Journal of Wildlife Diseases*.

[212] Callan RJ, Bunch TD, Workman GW, Mock RE (1991). Development of pneumonia in desert bighorn sheep after exposure to a flock of exotic wild and domestic sheep. *Journal of the American Veterinary Medical Association*.

[213] Miller MW, Williams ES, Barker IK (2001). Pasteurellosis. *Infectious Diseases of Wild Mammals*.

[214] Miller MW, Hobbs NT, Williams ES (1991). Spontaneous pasteurellosis in captive Rocky Mountain bighorn sheep (*Ovis canadensis* canadensis): clinical, laboratory, and epizootiological observations. *Journal of Wildlife Diseases*.

[215] Foreyt WJ (1989). Fatal *Pasteurella haemolytica* pneumonia in bighorn sheep after direct contact with clinically normal domestic sheep. *American Journal of Veterinary Research*.

[216] Rudolph KM, Hunter DL, Foreyt WJ, Cassirer EF, Rimler RB, Ward ACS (2003). Sharing of *Pasteurella* spp. between free-ranging bighorn sheep and feral goats. *Journal of Wildlife Diseases*.

[217] Ward ACS, Hunter DL, Jaworski MD (1997). *Pasteurella* spp. in sympatric bighorn and domestic sheep. *Journal of Wildlife Diseases*.

[218] Tomassini L, Gonzales B, Weiser GC, Sischo W (2009). An ecologic study comparing distribution of *Pasteurella trehalosi* and *Mannheimia haemolytica* between sierra nevada bighorn sheep, white mountain bighorn sheep, and domestic sheep. *Journal of Wildlife Diseases*.

[219] Kraabel BJ, Miller MW (1997). Effect of simulated stress on susceptibility of bighorn sheep neutrophils to *Pasteurella haemolytica* leukotoxin. *Journal of Wildlife Diseases*.

[220] Ward ACS, Weiser GC, Anderson BC, Cummings PJ, Arnold KF, Corbeil LB (2006). Haemophilus sommus (Histophilus Somni) in bighorn sheep. *Canadian Journal of Veterinary Research*.

[221] Coggins V, Mathews PE (1998). Field treatment of bighorns during pneumonia die-offs. *Biennial Symposium of the Northern Wild Sheep and Goat Council*.

[222] Kraabel BJ, Miller MW, Conlon JA, McNeil HJ (1998). Evaluation of a multivalent *Pasteurella haemolytica* vaccine in bighorn sheep: protection from experimental challenge. *Journal of Wildlife Diseases*.

[223] Cassirer EF, Rudolph KM, Fowler P, Coggins VL, Hunter DL, Miller MW (2001). Evaluation of ewe vaccination as a tool for increasing bighorn lamb survival following pasteurellosis epizootics. *Journal of Wildlife Diseases*.

[224] Rice JA, Carrasco-Medina L, Hodgins DC, Shewen PE (2007). *Mannheimia haemolytica* and bovine respiratory disease. *Animal Health Research Reviews*.

[225] Goodwin-Ray KA, Stevenson MA, Heuer C (2008). Effect of vaccinating lambs against pneumonic pasteurellosis under New Zealand field conditions on their weight gain and pneumonic lung lesions at slaughter. *Veterinary Record*.

[226] Unites States Department of the Interior, Bureau of Land Management (1998). *Revised Guidelines for Managment of Domestic Sheep and Goats in Native Wild Sheep Habitats*.

[227] DaMassa AJ, Wakenell PS, Brooks DL (1992). Mycoplasmas of goats and sheep. *Journal of Veterinary Diagnostic Investigation*.

[228] Ruffin DC (2001). Mycoplasma infections in small ruminants. *The Veterinary clinics of North America—Food animal practice*.

[229] Brogden KA, Rose D, Cutlip RC, Lehmkuhl HD, Tully JG (1988). Isolation and identification of mycoplasmas from the nasal cavity of sheep. *American Journal of Veterinary Research*.

[230] Lapinsky SE (2010). Epidemic viral pneumonia. *Current Opinion in Infectious Diseases*.

[231] Hansen DE, McCoy RD, Armstrong DA (1995). Six vaccination trials in feedlot lambs for the control of lamb respiratory disease complex. *Agri-Practice*.

[232] Vinten-Johansen P (2003). *Cholera, Chloroform, and the Science of Medicine: A Life of John Snow*.

[233] Lubin JH, Blot WJ, Berrino F (1984). Modifying risk of developing lung cancer by changing habits of cigarette smoking. *British Medical Journal*.

[234] Centre for Evidence-Based Medicine (2009). Oxford Centre for Evidence-Based Medicine. Levels of evidence. http://www.cebm.net/?o=1025.

[235] Zepeda C, Salman M, Ruppanner R (2001). International trade, animal health and veterinary epidemiology: challenges and opportunities. *Preventive Veterinary Medicine*.

[236] Budd L, Morag Bell, Brown T (2009). Of plagues, planes and politics: controlling the global spread of infectious diseases by air. *Political Geography*.

[237] Pybus MJ, Fenton RA, Lange H (1994). A health protocol for domestic sheep on forest grazing allotments in Alberta and British Colombia. *Biennial Symposium of the Northern Wild Sheep and Goat Council*.

